# Aging in the USA: similarities and disparities across time and space

**DOI:** 10.1038/s41598-020-71269-3

**Published:** 2020-08-31

**Authors:** Ana Lucia Abeliansky, Devin Erel, Holger Strulik

**Affiliations:** Department of Economics, University of Göttingen, Platz der Göttinger Sieben 3, 37073 Göttingen, Germany

**Keywords:** Biophysics, Signs and symptoms

## Abstract

We study biological aging of elderly U.S. Americans born 1904–1966. We use thirteen waves of the Health and Retirement Study and construct a frailty index as the number of health deficits present in a person measured relative to the number of potential deficits. We find that, on average, Americans develop 5% more health deficits per year, that men age slightly faster than women, and that, at any age above 50, Caucasians display significantly fewer health deficits than African Americans. We also document a steady time trend of health improvements. For each year of later birth, health deficits decline on average by about 1%. This health trend is about the same across regions and for men and women, but significantly lower for African Americans compared to Caucasians. In non-linear regressions, we find that regional differences in aging follow a particular regularity, akin to the compensation effect of mortality. Health deficits converge for men and women and across American regions and suggest a life span of the American population of about 97 years.

## Introduction

All humans age chronologically by a year each year. Biological aging, in contrast, is individual-specific and a 70-year-old can be as healthy as a 50-year-old. Biological aging is understood as the accumulation of damage to cells and tissues in the body and the gradual deterioration of bodily functions. Notwithstanding the idiosyncrasies in biological aging, there are some strong regularities discernible at the level of $\text{(sub-) }$ populations. In this study, we present some of these regularities for elderly United States (U.S.) Americans.

We measure individual health by constructing a frailty index, following the seminal work of Mitnitski et al.^1–3^. The index simply records the fraction of a large set of aging-related health conditions that is present in an individual. It has been shown that it does not matter for certain reproducible properties of the index, which particular health deficits are included in the unweighted index as long as there are sufficiently many (30 or more). The most important reproducible property in our context is that health deficits are log-linearly related to age, implying that they are accumulated at a constant rate (which has been estimated to be between 3 and 5%)^4,5^. See^5^ for methodological background and further discussion of the reproducible properties of the frailty index. The non-linear and convex pattern of deficit accumulation implies that deficits are not accumulated independently from each other and that the presence of many deficits is conducive to the development of more deficits over the next time increment (e.g. the next year). The intuition for this feature, as well as for the feature of independence from inclusion of specific health deficits in the frailty index, is that health deficits are connected to other health deficits. For example, hypertension is associated with the risk of stroke, heart diseases, kidney diseases, dementia, and problems of sleeping well and not being able to walk fast. This means that if a particular health deficit is missing from the list, its effect (on, for example, the probability of death) is taken up by a combination of other health deficits. This self-productive feature of the frailty index has a microfoundation in reliability theory^6^, and in a network theory of human aging^7^. The index thus captures in one number the biological aging process defined as the intrinsic, cumulative, progressive, and deleterious loss of function^8,9^.

The quality of the frailty index is mostly demonstrated by its predictive power for death at the individual level, and for mortality at the group level. The prediction of mortality can be so accurate that chronological age adds insignificant explanatory power when added to the regression^10^. The elimination of chronological age in the determination of aging and death is the ultimate goal of any successful theory of aging^8^. Other studies demonstrate the predictive power of the frailty index for the risk of institutionalization in nursing homes and becoming a disability insurance recipient^11–13^. Dalgaard and Strulik have integrated the frailty index into an economic life cycle theory of health, aging, and death^14^. The consideration of health deficits provides a biological foundation of health economic theory. It replaces the until then popular concept of unobservable health capital^15^ by an easily measurable concept established in gerontology and medical science and allows therewith for the development of quantifiable and testable health economic models. Applications consider, for example, the education gradient^16^, the long-term evolution of the age at retirement^17^, the gender gap in mortality^18^, the health gain from marriage^19^, and particular health behavior such as addiction^16^, self-control problems^20^, and adaptation to poor health^21^.

Another reason for the popularity of the frailty index, which has been used by now in hundreds of studies in gerontology and medical science, is that it can be easily compared across samples, datasets, and populations^5^. In the Discussion section we compare our results for Americans with those for Canadians^2,4^ and Europeans from 14 different countries^22,23^.

We investigate the differences in the level and rate of accumulation of health deficits between Caucasians and African Americans. These results relate to a literature arguing that access and quality of health care is biased against women and African Americans^24–27^. We use year-of-birth trends in the frailty index to uncover long-run trends in health improvements of Americans. Other studies on progress in human health focused on improvements in nutrition and stature^28,29^ and mortality^30^. Vaupel concludes that human senescence has been delayed by a decade in the sense that levels of mortality that used to prevail at age 70 now prevail at age 80, and levels that used to prevail at age 80 now prevail at age 90^31^. Dalgaard et al. construct aggregate health deficit indices for the working-age population of 191 countries and show that, over the last quarter of century, the workforce did not age in physiological terms, although it got chronologically older^32^. Here, we focus on regularities in the development of average health deficits in cohorts of subpopulations. As the number of average health deficits increases with age, the variance of health deficits also increases in a specific way, which has been explored in related literature^12,33^; see^34^ for a discussion of implications on health inequality in the framework of optimal health insurance and retirement policy.

## Data and empirical strategy

For our analysis, we used the Health and Retirement Study RAND HRS Longitudinal File 2016 (V1). This data was compiled by the RAND Center of the Study of Aging, with funding from the National Institute on Aging and the Social Security Administration. We used the public use dataset and considered waves 1 to 13. The first wave took place in 1992, the second one in 1993/1994, and wave 3 in 1995/1996. From then onwards the survey continued biennially. We considered respondents aged 50 and above at the time of their first interview. Because a significant share of the oldest old individuals show “super healthy” characteristics, we focus on individuals aged 90 and below to avoid selection effects. However, as shown in the Appendix, we obtain similar results when we abandon the age cutoff and when we apply an even stricter cutoff at age 85.

In line with our definition of aging as the (yearly) accumulation of health deficits, we created a frailty index for each individual, following the methodology developed in^1^. We considered symptoms, signs, and disease classifications to construct the index. A summary of all 38 deficits considered is given in the Appendix (Table A1).

The frailty index is computed as the proportion of deficits that a respondent suffers from out of the number of potential health deficits. We coded multilevel deficits using a mapping to the Likert scale in the interval 0–1. In case of missing data for an individual on one or several deficit(s), we constructed the frailty index based on the available information (i.e. if for a particular individual data were not available for *x* potential health deficits, the sum of the observed health deficits was divided by $38-x$). From the surveyed individuals, we kept only those with information on at least 30 health deficits. Due to missing values in the creation of the frailty index or because of the lack of sufficient deficits to reach the 30-item minimum, we lost less than 6% of the observations of the initial dataset. Further, we dropped observations where the region of residence and/or the place of birth was missing, besides those born outside of the U.S.. By excluding migrants we focus on a more homogenous group of individuals exposed to the U.S. American health environment for their whole life. The reduced dataset contains 177,502 observations. In the first core sample, the HRS includes three oversamples. The sample is designed to increase African American and Hispanic individuals, and residents living in the state of Florida. The dataset includes compensatory weights. However, since the dataset is cleaned according to the limitations described above, the original structure of the sample is not preserved. Thus, sample weights will be ignored in the main analysis. This approach is also supported by Yang and Lee^35^, who also used the HRS dataset to construct a frailty index, refraining from using sample weights. They argue that it will not lead to significantly different results and they follow the recommendations of Winship and Radbill^36^.

Summary statistics are shown in Table A3 in the Appendix. Individuals are born between 1904 and 1966 with an average year of birth of 1936. On average, elderly Americans display a frailty index of about 20%. Women are on average more frail than men and African Americans are more frail than Caucasians. The difference between the number of all individuals and the sum of Caucasians and African Americans results from the presence of individuals of other ethnicities (Hispanics, Asians, etc). The sample contains 16,486 more female than male observations.

We estimate the log-linear relationship between age and health deficits with the following equation:

1 $$\begin{aligned} \log D_{iw}=\beta +\alpha \cdot age_{iw}+\sum _{t=1}^{T-1}\gamma _t \cdot yob_{it}+\varepsilon _{iw} \end{aligned}$$

where $D_{iw}$ is the frailty index, *i* represents the individual, *w* the wave, *age* represents the age at the end of the interview, *t* refers to the year of birth and $\varepsilon$ is the error term; *yob* is a set of dummy indicators which are one when *t* equals the year of birth of individual *i* (and the $\gamma$’s are the associated year-of-birth fixed effects); and *T* if is the last year of birth in the respective sample. Subsequently, when we speak of accumulated health deficits, we always refer to them in relative terms, i.e. relative to potential deficits, as measured by the frailty index $D_{iw}$. We estimate (1) separately for gender given that previous research showed that men and women age differently^2,22^. Since we have broad information on ethnicity, we also estimated the model for two subsamples (African American and Caucasian). When we estimate the same relationship but using fixed effects, we assume that the error term $\varepsilon _{iw}$ is now composed of $\mu _i$ and $u_{iw}$, where the unobserved individual effects $\mu _i$ are correlated with the regressors (the time-invariant variables are now dropped since they are perfectly collinear with the fixed effects) and $u_{iw}$ is the idiosyncratic error term. Instead, with the Mundlak approach^37^, we assume that $\mu _i$ (still unobserved) are not correlated with the regressors (i.e. the assumption in a random effects model) and we add the individual-time means of the time-changing variables. The estimated equation is given by $\log D_{iw}=\beta +\alpha \cdot age_{iw}+ \beta \cdot \bar{age_i} + \mu _i + u_{iw}$, in which $\bar{age_i}$ is the mean age of individual *i*. The Mundlak model is essentially a random effects estimator with the addition of the individual-means of the time-changing covariates. Mundlak^37^ has shown that the estimates of the time changing variables of his approach should be comparable to those of a fixed effects estimator.

The log-linear equation implies that health deficits accumulate exponentially with age, $D=R\mathrm{e}^{\alpha \cdot age}$, with $R=\mathrm{e}^{\beta }$, akin to the Gompertz law of mortality^38^.

## Panel estimation results

### Similarities and disparities of individual aging

Results from log-linear regressions for women and men are shown in Table 1. We first focus on individual aging and thus the preferred estimation method includes individual fixed-effects to account for unobserved heterogeneity at the individual level. Results are shown in columns 1–3. In line with previous research, we find that the age coefficient is higher for men than for women and the constant is lower for men. These differences are mild but statistically significant. For the whole sample, the frailty index for men increases by 5.66 ($\pm 0.12$) percent and the one for women by 5.04 ($\pm 0.16$) percent by each additional chronological year of age. This means that men accumulate health deficits (mildly) faster but start out at a lower level of health deficits. A different view on the same results emphasizes commonalities of the aging process: on average, elderly Americans develop about 5% more health deficits from one birthday to the next.

The regional fixed-effects are mostly insignificant. Since we control for individual fixed-effects, the regional coefficients pick up the health impact of moving. The omitted region is the Northeast. Apparently, moving to the South is associated with fewer health deficits for both men and women. The causality, however, is unclear. It may well be that richer and thus healthier individuals are more motivated to move to a warmer climate after retirement. For Caucasians of both genders, the age coefficient is higher and the constant is lower than for African Americans, implying that initially healthier Caucasians age faster than African Americans.

Although attrition rates are low in the HRS^39^, we performed a variable addition test, as suggested by^40^ and as employed by^41^. We have added as an extra variable whether a person is present in the next wave or not. Although the added variable is statistically significant, we find no evidence of attrition affecting our results. Tables A6 and A7 in the Appendix show these results. Moreover, we have performed two other robustness tests. The first is to reduce the maximum age from 90 to 85 and the second one to eliminate the age restriction. The results can be found in Tables A8–A11 in the Appendix and they do not differ significantly from those of Table 1.

**Table 1:** Panel estimation results.

	(1)	(2)	(3)	(4)	(5)	(6)
Women						
Age	0.0504$$***$$	0.0457$$***$$	0.0517$$***$$	0.0504$$***$$	0.0457$$***$$	0.0517$$***$$
	(0.00160)	(0.00127)	(0.00177)	(0.00161)	(0.00127)	(0.00178)
Midwest	$-$0.0406	0.149$*$	$-$0.0757	$-$0.0409	0.149$*$	$-$0.0760
	(0.0431)	(0.0804)	(0.0501)	(0.0430)	(0.0804)	(0.0499)
South	$$-$$0.0652$$**$$	$$-$$0.0237	$$-$$0.0691$$*$$	$$-$$0.0655$$**$$	$$-$$0.0235	$$-$$0.0697$$*$$
	(0.0321)	(0.0554)	(0.0365)	(0.0321)	(0.0555)	(0.0363)
West	$-$0.0547	0.0175	$-$0.0750	$-$0.0553	0.176	$-$0.0757
	(0.0444)	(0.125)	(0.0479)	(0.0443)	(0.0125)	(0.0478)
Year of birth				$$-$$0.00989$$***$$	$$-$$0.0104$$***$$	$$-$$0.0152$$***$$
				(0.00232)	(0.00267)	(0.00232)
Mean Age				$$-$$0.0419$$***$$	$$-$$0.0434$$***$$	$$-$$0.0455$$***$$
				(0.00417)	(0.00438)	(0.00427)
Constant	-5.185$$***$$	-4.607$$***$$	-5.350$$***$$	16.94$$***$$	18.58$$***$$	27.10$$***$$
	(0.109)	(0.0972)	(0.122)	(4.670)	(5.407)	(4.664)
Sample	All	African American	Caucasian	All	African American	Caucasian
Method	FE	FE	FE	Mundlak	Mundlak	Mundlak
Observations	96414	18224	75442	96414	18224	75442
Men						
Age	0.0566$$***$$	0.0546$$***$$	0.0569$$***$$	0.0565$$***$$	0.0545$$***$$	0.0568$$***$$
	(0.00122)	(0.00148)	(0.00128)	(0.00123)	(0.00148)	(0.00128)
Midwest	$-$0.0332	$-$0.173	$-$0.00692	$-$0.0331	$-$0.168	$-$0.00742
	(0.0406)	(0.118)	(0.0449)	(0.0406)	(0.118)	(0.0450)
South	$$-$$0.112$$***$$	$$-$$0.175$$**$$	$$-$$0.0985$$**$$	$$-$$0.112$$**$$	$$-$$0.177$$**$$	$$-$$0.0987$$**$$
	(0.0361)	(0.0802)	(0.0419)	(0.0362)	(0.0786)	(0.0419)
West	$$-$$0.117$$**$$	$$-$$0.224$$**$$	$$-$$0.105$$*$$	$$-$$0.115$$**$$	$$-$$0.207$$**$$	$$-$$0.106$$*$$
	(0.0507)	(0.107)	(0.0552)	(0.0511)	(0.105)	(0.0555)
Year of birth				$$-$$0.00835$$***$$	$$-$$0.00172	$$-$$0.0130$$***$$
				(0.00164)	(0.00227)	(0.00177)
Mean Age				$$-$$0.0461$$***$$	$$-$$0.0392$$***$$	$$-$$0.0497$$***$$
				(0.00273)	(0.00325)	(0.00295)
Constant	$$-$$5.725$$***$$	$$-$$5.308$$***$$	$$-$$5.811$$***$$	13.46$$***$$	0.408	22.62$$***$$
	(0.0939)	(0.0977)	(0.101)	(3.299)	(4.548)	(3.572)
Sample	All	African American	Caucasian	All	African American	Caucasian
Method	FE	FE	FE	Mundlak	Mundlak	Mundlak
Observations	80042	11970	65684	80042	11970	65684

Robust standard errors clustered at the year of birth level in parenthesis. All columns include regional fixed effects, the baseline category is the region “Northeast”, columns 1–3 further include individual fixed effects. Columns 4–6 further control for the year of birth and the (time) means of the time changing variables. $$*$$$$p<0.10$$, $$**$$$$p<0.05$$, $$***$$$$p<0.01.$$.

**Figure 1:**
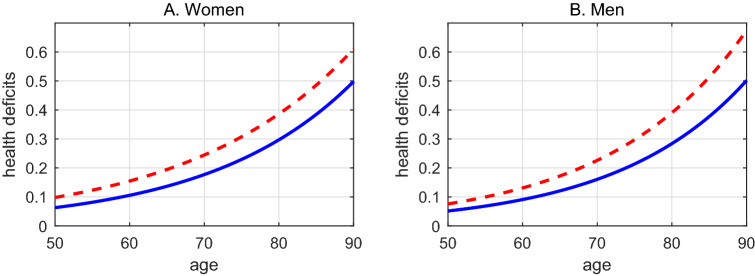
Frailty index by age. Solid (blue) lines: predicted health deficits by age for Caucasians. Red (dashed) lines: predicted health deficits by age for African Americans. Health deficits measured by the frailty index.

Figure 1 visualizes the estimation results by showing the predicted health deficits by age implied by the point estimates from column (2) and (3) in Table 1. It reveals a feature that is hard to discern from the estimates in Table 1, namely that Caucasians (represented by blue solid lines), at any age, have developed fewer health deficits than African Americans (represented by red dashed lines). On average, African Americans display a 7% points higher frailty index and the difference between African Americans and Caucasians becomes larger as individuals grow older, in particular for men.

### Aging of cohorts

**Figure 2:**
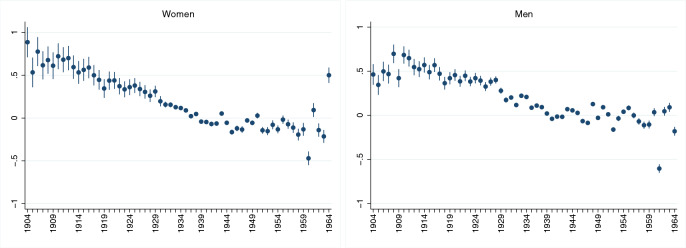
Year of birth fixed effects. Year of birth fixed effects retrieved from the Mundlak regressions (Table 2, column (4)).

We next look at cohort-effects on aging by including year-of-birth fixed effects. This implies that we have to drop the individual fixed effects. In order to still control for individual heterogeneity (of the time-variant variables), we follow the Mundlak approach^37^. The Mundlak estimator is composed of a random effects regression that includes time averages (at the individual level) of the time-changing variables. Results of the Mundlak specification are presented in columns 4–6 in Table 1. The Mundlak term ‘Mean Age’ is statistically significant in all regressions, thus reinforcing the results of the Hausman test that there is heterogeneity at the individual level (correlated with the force of aging). The rather long tables containing all year of birth dummies are included in the Appendix (Tables A4 and A5). The main takeaway from these regressions is that the year of birth coefficient is always significant and that its size declines almost linearly in the year of birth. This feature is visualized in Fig. 2. The reference year of birth is 1934. The declining trend is clearly visible and from the early 1910s to the late 1940s where it appears to be linear. From the 1950s onwards, the trend seems to decline somewhat. However, the impression of linearity is also blurred by the high variation of the the year-of-birth effect at lowest and highest years of birth. This variation can be attributed to the low number of observations at both ends of the year-of-birth range, as shown in Table A2 in the Appendix.

Encouraged by the (almost-) linear decline of the year-of-birth coefficient, we replaced the year-of-birth dummies by a constant year of birth trend. Results are shown in columns 4–6 of Table 1. Considering the whole sample, we observe that women have about 1% fewer health deficits per later year of birth (0.99 $\pm 0.23)$. For men, the health trend is slightly but insignificantly smaller than for women (at $0.84 \pm 0.16$% per year).

The result, however, is refined when we split the sample by ethnicity. We then find a substantially faster health trend for Caucasian women ($1.53 \pm 0.27$%) and men ($1.32 \pm 0.18$%) and a substantially slower health trend for African Americans. For African American men, the trend estimate differs insignificantly from zero, suggesting that this group did not benefit from generally improving health status in the elderly population.

In Tables A4 and A5 in the Appendix we provide results of the year of birth trend interacted with region fixed effects. For men and women the coefficients of the interaction are similar to the coefficient of the general year of birth trend and highly significant. This means that the observed decline of health deficits is not specific to a region - but observable and similar in size across all regions.

**Figure 3:**
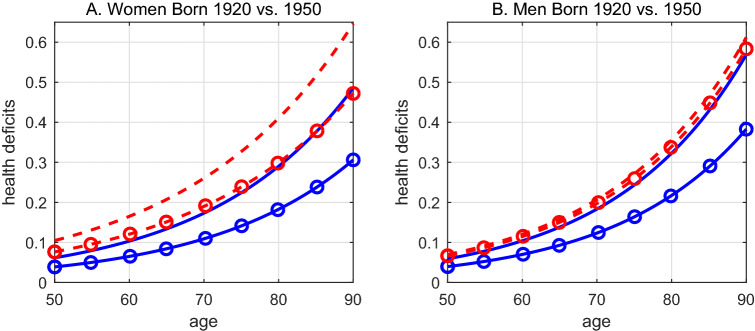
Aging of 1920 vs. 1950 Cohort. Predicted aging process from estimates in columns (5) and (6) of Table 2: Caucasians (blue solid lines) and African Americans (red dashed lines) born 1920 (no markers) and born 1950 (circles).

Figure 3 shows the predicted aging process of Caucasians (blue solid lines) and African Americans (red dashed lines) born 1920 (no markers) and born 1950 (circles). The later born cohorts of Caucasian women and men are predicted to display significantly fewer health deficits at any age. On average, thirty years of later birth shift the age trajectory of health deficits down by about 7 percentage points. The shift, however, is not parallel, the health gain from later birth increases in age. For example, the frailty index that the 1920-cohort of women displayed at age 60 (age 75) is predicted for the 1950 cohort at age 67 (age 89). Caucasian men experience similar albeit slightly smaller health gains from late birth. Significant improvements in health are also predicted for African American women. For example, a frailty index of 0.21, displayed at age 65 of the 1920-cohort, is predicted for the 1950-cohort at age 72. At that age, the 1950-cohort of African American women arrives at about the same frailty index as the 1920-cohort of Caucasian women. The 1950-cohort of Caucasian women, in contrast is significantly healthier, and displays a frailty index of 0.21 only at age 82. African American men born 1920 differed less from Caucasians than their female counterparts. However, they did not benefit from generally improving health and the 1950-cohort is still at any age less healthy than Caucasians born in 1920.

Figure A1 in the Appendix provides a different view on the same information. It shows the health deficits predicted by year of birth for a 75 year old person, separately for gender and ethnicity. Again, blue (solid) lines represent Caucasian and red (dashed) lines African Americans. The figure shows the steady improvement of health status with year of birth. For Caucasians, the frailty index declined from a level of about 0.25 for the 1920 cohort to a predicted level below 0.15 for the 1960 cohort. The frailty index that Caucasian women had in 1920 is reached by African American women of the 1951-cohort.

## Nonlinear regression results

### Basic results

In this section, we abandon the log-linear specification and estimate a quasi-exponential relationship according to the Gompertz–Makeham structure. This approach is motivated by the conceptual similarity of aging understood as health deficit accumulation and aging understood as increasing mortality^2^. Makeham proposed to add a constant (capturing non aging-related death) to the Gompertz model of mortality^42^, resulting in a log-linear association of the rate of mortality with age. The Gompertz-Makeham model turned out to be very successful in predicting death at the population level and its parameters have been estimated with great precision^8,43,44^.

Given the close relationship of the frailty index with the mortality rate and its predictive power for death^10^, it seems reasonable that the frailty index exhibits a similar association with age as the mortality rate. This view is also supported by theoretical models of aging based on depletion of redundancy in reliability theory^6^ and based on health deficit transitions in networks^7^. This implies that, if health deficits exhibit the same functional association with age as mortality, then ignoring the Makeham-term could bias the results. Analogously to the mortality studies, the Makeham-term captures environmental factors that influence health deficits independently from age such as regional-specific health care institutions that determine the access and quality of health care or the age-independent discrimination in health care with respect specific demographic groups.

The feature that the Gompertz-Makeham model needs to be estimated with non-linear regression prevents the inclusion of individual fixed effects (as in the linear Gompertz regressions of the previous section). The inclusion of such high-dimensional individual fixed effects reduces substantially the degrees of freedom such that we would run into an incidental parameter problem and the non-linear regression algorithm would fail to converge. We thus shift the focus in this section from the aging of individuals and cohorts to the aging of U.S. American sub-populations.

Using the pooled sample, we estimated the accumulation of health deficits with the following model:

2 $$\begin{aligned} D_{i}=A+R\cdot \mathrm{e}^{\alpha \cdot age _{i}}+\epsilon _{i}, \end{aligned}$$

separately for gender and ethnicity and later also separately for the main U.S. American regions. For linguistic convenience, we refer to *A* as the Makeham term and $\alpha$ and *R* as Gompertz terms.

**Table 2:** Results: nonlinear least squares.

	(1)	(2)	(3)	(4)	(5)	(6)
A	0.0525$$***$$	0.138$$***$$	0.112$$***$$	0.165$$***$$	0.0223$$*$$	0.108$$***$$
	(0.0100)	(0.00384)	(0.0215)	(0.0153)	(0.0130)	(0.00478)
R	0.0134$$***$$	0.00209$$***$$	0.00662	0.00589$$*$$	0.0199$$***$$	0.00329$$***$$
	(0.00328)	(0.000422)	(0.00509)	(0.00312)	(0.00503)	(0.000667)
$$\alpha$$	0.0337$$***$$	0.0534$$***$$	0.0405$$***$$	0.0429$$***$$	0.0303$$***$$	0.0493$$***$$
	(0.00245)	(0.00220)	(0.00809)	(0.00557)	(0.00246)	(0.00218)
Sample	All	All	African American	African American	Caucasian	Caucasian
Gender	Men	Women	Men	Women	Men	Women
$R^{2}$	0.0892	0.0900	0.0560	0.0682	0.1070	0.1153
Observations	80823	97321	12079	18273	66341	76282

Robust standard errors in parenthesis. $$*$$$$p<0.10$$, $$**$$$$p<0.05$$, $$***$$$$p<0.01$$.

**Figure 4:**
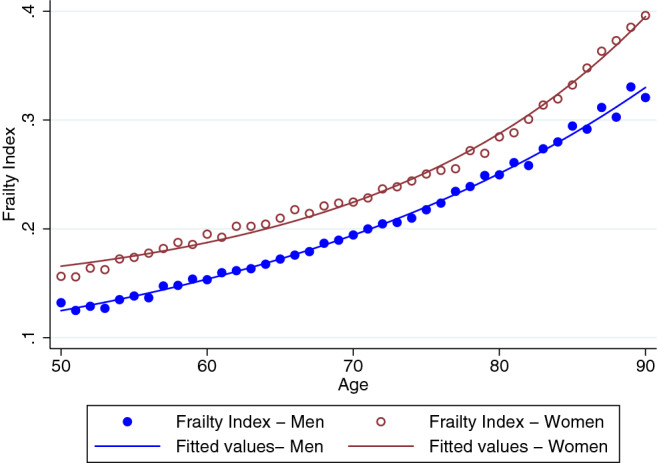
Nonlinear least squares results and binned data points.

Regression results are shown in Table 2. The Makeham term is statistically significantly different from zero and larger for women than for men as well as larger for African Americans than for Caucasians. It is largest for African American women. As indicated by the $R^2$-values, the explained variation of health deficits is rather low. However, this feature simply reflects the fact that aging is highly idiosyncratic. At the population level, the accumulation of health deficits with age looks almost deterministic. This is shown in Fig. 4 where the predicted health deficits from column (1) and (2) in Table 2 are confronted with the actual mean frailty index by age. Averaging over age takes out most of the idiosyncrasies and the prediction fits the data reasonably well. This feature is also reflected in Table A14 in the Appendix, which shows an $R^2$ above 0.99 when the data is binned in annual age groups. The estimated coefficients in the binned regressions differ insignificantly from the results for the nonbinned data. As an additional robustness test, Tables 12 and 13 in the Appendix show the results without age restriction and for a lower cutoff age of 85. Again, results are very similar to those from the basic regressions of Table 2.

**Figure 5:**
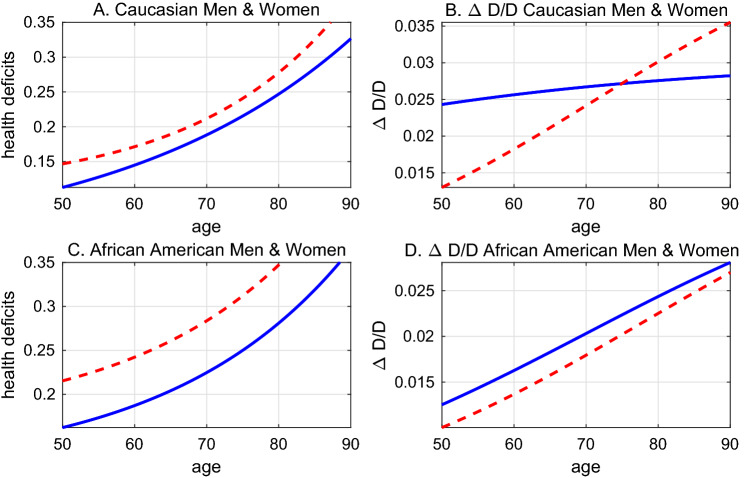
Health deficit accumulation and speed of aging. Left-hand side: predicted health deficits by age. Right-hand side: predicted speed of aging $\Delta D/D$. Solid (blue) lines: men. Red (dashed) lines: women.

The estimated coefficient of the age-term ($\alpha$) in Table 2 is larger for women than for men. This seemingly suggests a contradiction to the findings from log-linear regression, where the speed of aging of men was slightly higher. The speed of aging, however, can no longer be read off from the age-coefficient. It is is given by $\dot{D}/D = \alpha R\mathrm{e}^{\alpha t}/(A+R\mathrm{e}^{\alpha t})$ and varies with age for $A\not =0$. Figure 5 illustrates the regression results from column 3–6 of Table 2. The panels on the left-hand side confirm the earlier result that women (represented by red dashed lines) are predicted to display more health deficits than equally aged men (represented by blue solid lines). The panels on the right hand side show the implied speed of aging, i.e. the rate at which new health deficits are accumulated. For Caucasian men, for whom *A* is close to zero, the speed of aging is almost constant. For the other groups, the speed of aging is increasing with age. Compared to women, the speed of aging is greater for African American men and for Caucasian men below 75, which largely confirms the earlier results.

### Regional disparities

We next focus on aging in the four main U.S. American regions classified in the HRS Data: Northeast, Midwest, South, and West. Since there are too few African Americans in some regions for consistent estimates, we only kept the distinction between men and women and focused on the sample split by regions instead. Table 3 shows the results from nonlinear regressions. The Makeham term is significantly positive for women of all regions and everywhere greater than for men, suggesting that the potential health care bias obtained above for the whole country is also present in every region, with insignificant differences between regions. The estimated $\alpha$-coefficients differ across regions. Since the $\alpha$ estimates are quite precise, this suggests that people age faster in some regions than others. Interestingly, regions that display a high $\alpha$-coefficient simultaneously display a low value of the *R*–coefficient. Since $R+A$ captures initial health deficits at age 50 and since *A* does not systematically vary across regions (at least for women), the results suggest that there is regional convergence: people age faster in regions where they are initially healthier.

**Table 3:** Nonlinear least squares by region.

	Northeast	Midwest	South	West
Women				
A	0.136$$***$$	0.127$$***$$	0.134$$***$$	0.130$$***$$
	(0.00919)	(0.00701)	(0.00779)	(0.00661)
R	0.00223$$**$$	0.00215$$***$$	0.00415$$***$$	0.00119$$**$$
	(0.00112)	(0.000767)	(0.00128)	(0.000507)
Alpha	0.0517$$***$$	0.0533$$***$$	0.0464$$***$$	0.0599$$***$$
	(0.00543)	(0.00387)	(0.00328)	(0.00471)
Observations	18443	26346	45008	19882
$R^{2}$	0.081	0.105	0.083	0.101
Men				
A	0.0683$$***$$	0.0355$$*$$	0.0230	0.0879$$***$$
	(0.0146)	(0.0189)	(0.0248)	(0.00990)
R	0.00588$$*$$	0.0152$$**$$	0.0280$$**$$	0.00266$$*$$
	(0.00306)	(0.00653)	(0.0113)	(0.00140)
Alpha	0.0424$$***$$	0.0328$$***$$	0.0269$$***$$	0.0500$$***$$
	(0.00548)	(0.00425)	(0.00376)	(0.00574)
Observations	14246	21987	37199	17440
$R^{2}$	0.108	0.110	0.079	0.088

Robust standard errors in parenthesis. $$**$$$$p<0.05$$, $$***$$$$p<0.01$$.

**Figure 6:**
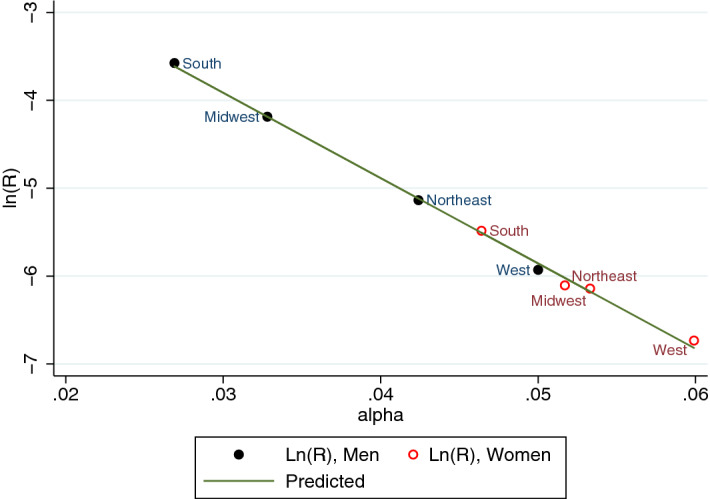
Compensation effect.

The negative relationship between the Gompertz parameters is known in the demographic literature as Strehler-Mildvan-correlation, or “compensation effect of mortality”^6,45^. There, sub-populations with lower initial mortality display a larger increase of mortality with age such that there exists a common age at which all sub-populations display the same mortality rate. Figure 6 shows that a similar regularity is also visible for the Gompertz parameters of the frailty index regressions (*R* and $\alpha$). Men from the South and Midwest are initially, at age 50, less healthy than men from the West but develop new health deficits at a slower pace. A similar relation exists for women. Taken together, the picture suggests a linear relationship between $\alpha$ and $\log R$.

In order to explore this relationship further, we followed^2^ and regressed $\log R$ on $\alpha$ across regions and gender:

3 $$\begin{aligned} \log R_{rg}=\beta - T \cdot \alpha _{rg}, \end{aligned}$$

in which $R_{rg}$ and $\alpha _{rg}$ are the regional- and gender-specific parameter estimates from Table 3. Results are shown in Table 4. The coefficient for *T* is estimated to be close to 97 in column (1). The next column controls for gender by adding a female dummy variable. The dummy variable is not significant and the point estimate for *T* increases by two units but differs insignificantly from the estimate of column (1). Since the female dummy is not statistically significant, we prefer the specification from column (1) because of the higher degrees of freedom.

The compensation effect of mortality has been used to infer the life span of a population^6^. In contrast to life-expectancy, life span is conceptualized as a time- and situation-invariant, in our specific case, “the” life-span of Americans, regardless or provenance and gender. Defining human life span as the maximum attainable age at death, as suggested in many general dictionaries and many older contributions in biology is misleading^46^. Empirically it has been refuted by the observation that maximum age at death has been continuously on the rise for at least 140 years^47^. Instead, biogerontologists have suggested to define life span as the age at which the Gompertz-Makeham mortality-trajectories intersect. If such a common intersection exists, it identifies a constant that is shared by all members of the population independently from environmental and genetic characteristics. This constant is the age at which all members of a population are predicted to display the same mortality rate. It has been suggested to apply the same logic to the accumulation of health deficits, which exhibit a similar regularity^2,3^. To see why the parameter *T* in (3) identifies a population-specific constant, insert equation (3) into equation (2) to obtain $D_i-A=M\mathrm{e}^{-\alpha _{rg}(age_i-T)}$, with $M\equiv e^{\beta }$. Thus, controlling for aging-independent health *A*, the data predicts that on average, U.S. American men and women from all regions have developed the same frailty index at age *T*, which suggest that the life span of American is about 97 years.

**Table 4:** Compensation effect.

	(1)	(2)
T	$$-$$97.10$$***$$	$$-$$99.09$$***$$
	(2.126)	(3.121)
$$\beta$$	$$-$$1.001$$***$$	$$-$$0.939$$***$$
	(0.0990)	(0.123)
female		0.0569
		(0.0641)
Observations	8	8
$R^{2}$	0.997	0.998
Adjusted $R^{2}$	0.997	0.997

Standard errors in parentheses.

$$*$$$$p<0.10$$, $$**$$$$p<0.05$$, $$***$$$$p<0.01$$.

## Discussion and conclusions

Using data from the Health and Retirement Study^48^, we showed that elderly Americans born between 1904 and 1964 develop on average about 5% more health deficits from one birthday to the next. The exponential accumulation of health deficits confirms results from earlier longitudinal studies of other populations, which found a rate of deficit accumulation of about 4.5% for Canadians^4^) and of about 2.5%, on average, for 14 European countries^22^). In comparison, Americans appear to age (somewhat) faster, which, however, does not necessarily imply that they display more health deficits for any given age. This conclusion would only be compelling if the constant in the Gompertz regressions would also be larger for Americans, which is not the case, in comparison with Europeans^22^. A convex path of deficit accumulation for Americans has also been found by^35^ who focus on a quadratic association between the frailty index and age. The exponential (or convex) accumulation of health deficits suggests that biological aging is a self-productive process, in which the presence of many health deficits is conducive to the faster development of new deficits^49^. It supports theories of aging that are build on the interdependence of health deficits such as reliability theory^6^ and network theories of aging^7^.

Our study confirms the result of several previous studies that women, at given age, display more health deficits than men^4,35,50,51^, see^52^ for a review and meta study, and that men develop new health deficits faster than women^2,3,22,53^. The feature that systems that are initially less damaged, age at faster rate is a natural outcome of the reliability theory of aging^6^.

Since it is well known that mortality is lower for (American) women than for men and since the frailty index has been shown to be highly predictive of mortality, our study indirectly contributes to the morbidity-mortality paradox. The paradox is captured in the related literature by estimates of a stronger effect of the frailty-index score on mortality for men^4,33,34,50,51,54^. Potential explanations of the paradox within the frailty-index paradigm include the features that women suffer more often from non-lethal health deficits and that women visit doctors more often and report more diagnoses of deficits. These explanations have also been discussed in the rich literature on the morbidity-mortality paradox outside the frailty-index paradigm, which also discusses biological and genetic gender differences, explanations based immune system responses, hormones, disease patterns, and gender differences in health behavior as potential explanations^18,55–59^.

In cohort analysis we found an almost constant trend at which biological aging improves over time. For every year of later birth, elderly Americans display about 1% fewer health deficits at any age, implying, for example, that a 70-year-old born in 1960 is predicted to be about as healthy as a 60-year-old born in 1910. The rate of progress in individual health is the same across the main U.S. American regions (Northeast, Midwest, West, South) and insignificantly faster for women than for men. It stands to reason to interpret the steady health trend as access to better health care and medical progress, broadly interpreted, including, for example, better knowledge about the health-damaging impact of smoking. The study by Yang and Lee^35^ also investigated cohort effects albeit only for four distinct and several birth-years comprising cohorts born 1924–1947 (while we consider 60 cohorts born 1904–1964). For the coarse-grained cohort analysis the study found that later born cohorts had higher levels and steeper growth rates in frailty than earlier cohorts. However, since this result was obtained controlling for several other factors, it is not necessarily inconsistent with our result of an almost constant positive trend. It may well be that the generally positive trend is picked up by trending factors such as education.

In related work, a similar but higher health trend has been estimated for 14 European countries^22^. Europeans displayed 1.4–1.5 fewer health deficits per later year of birth, with insignificant differences between men and women and between countries. The lower trend for Americans suggests that Americans benefitted to a lower degree from perpetual medical progress and that their health diverges over time from that of Europeans. While the static inefficiency of the American health system is well-known^60–62^ the feature of dynamic inefficiency (lower rate of improvement) is perhaps less well known. Because of knowledge diffusion, we would expect that medical knowledge advances at the same if not faster pace in a technological frontier country such as the U.S. Moreover, economic theory suggests that we should observe convergence of similar systems such that initially backward (more inefficient) systems improve temporarily at higher rates^63^. The observation of a diverging health trend between Americans and Europeans is consistent with the more familiar phenomena that life expectancy improves in-sync with healthy life expectancy^64^ and that life expectancy increased at a slower rate in the U.S. than in Europe. From 1950 to 2000, life expectancy at birth increased by 8.6 years (from 68.2 to 76.2) in the U.S. and by 11.3 years (from 67.0 to 78.3) in Western Europe^65^.

However, when we divide the sample by ethnicity, the trend results become substantially refined. We then find that the frailty index for Caucasian Americans improves at a rate of 1.3–1.5% per year of birth, a rate that differs insignificantly from the European estimates. The health trend of African Americans, in contrast, is substantially slower. In particular, elderly African American men seem not to benefit from generally improving health status in the elderly population. This means that we observe not only static inequality, confirming results in^35^, i.e., for given age, a frailty index that is significantly higher for African Americans than for Caucasians, but also dynamic inequality, i.e. health disparities between Caucasians and African Americans that become larger over time. African Americans do not participate fully in health advances that are experienced at about the same rate by Europeans and Caucasian Americans. It should be noticed, however, that the elderly Americans in our study were not much affected by the opioid epidemic. The evidence compiled in^66^ shows that the opioid epidemic is particularly prevalent among young and middle-aged non-college educated Caucasians. Deteriorating health in this group counteracts ethnic disparities and it remains to be seen whether a widening ethnicity gradient of the frailty index will be a robust phenomenon for future generations of elderly Americans.

In non-linear regressions (akin to the Gompertz-Makeham law of mortality) we also find non-aging related health deficits to be larger for women and African Americans than for Caucasian men, which corroborates previous findings on the presence of biased access to health care^24–27^. Exploring differences in biological aging between the major regions of the U.S., we find that individuals are, on average, healthiest in the West and least healthy in the South. With increasing age, however, these differences converge such that there exists an age at which all Americans who survived to this age are predicted to be equally (un-) healthy, irrespective of gender or provenance. This age, which has been suggested to be associated with life span, is estimated as 97 $\pm 2$ years. It differs insignificantly from previous estimates for Canadians (94 $\pm 2$ years)^2^ and is somewhat lower than previous estimates for Europeans (102 $\pm 2.6$ years)^22^.

The log-linear health deficit model implies that health deficits are accumulated exponentially with increasing age *t*, $D(t)=\mathrm{e}^{\alpha t}$. The first derivative of this expression provides the increase of health deficits by age. It can be written as $\mathrm{d}D(t)/\mathrm{d}t= \alpha D(t)$. This means that unhealthy individuals, i.e. individuals who display already many health deficits, develop more new health deficits than healthy individuals. A popular model in health economics is based on the idea of health capital accumulation^15^. There, the assumption of health depreciation at a (potentially age-dependent) rate $\delta (t)$ implies that, at any age *t*, individuals lose health capital $\delta (t) H(t)$ through health capital depreciation, which means that healthy individuals who are equipped with a high health capital stock *H*(*t*), lose more health capital through health depreciation than unhealthy individuals with low *H*(*t*). If health capital is inversely related to the number of health deficits present in a person, which appears to be a plausible assumption, the health capital model predicts the opposite of the health deficit model. Then, the evidence provided in our study contradicts the health capital model because it supports the health deficit model for U.S. Americans. It confirms earlier studies, which found a similar (quasi-) exponential growth of health deficits for Canadians and Europeans.

## Supplementary information

Supplementary Information

## Data Availability

The raw data of the study is from the Health and Retirement Study (RAND HRS 2014 Fat File (V2A)), which is a public use dataset. It was produced and distributed by the University of Michigan with funding from the National Institute on Aging (grant number NIA U01AG009740). Ann Arbor, MI, (September 2019). RAND HRS 2014 Fat File (V2A) was produced by the RAND Center for the Study of Aging, with funding from the National Institute on Aging and the Social Security Administration. Santa Monica, CA (September 2019). The HRS (Health and Retirement Study) is sponsored by the National Institute on Aging (grant number NIA U01AG009740) and is conducted by the University of Michigan.
