# Supplementary material for: Aging in the USA: similarities and disparities across time and space
Source: Sci Rep. 2020 Aug 31;10:14309. doi: 10.1038/s41598-020-71269-3 (PMC7458930; doi:10.1038/s41598-020-71269-3)
Supplement: Supplementary file 1 — Supplementary Information [file 41598_2020_71269_MOESM1_ESM.pdf]

Aging in the USA: Similarities and Disparities Across Time and Space

Appendix

Ana Lucia Abeliansky[1]
Devin Erel[2]
Holger Strulik[3]

[1]University of Göttingen, Department of Economics, Platz der Göttinger Sieben 3, 37073 Göttingen, Germany.
[2]University of Göttingen, Department of Economics, Platz der Göttinger Sieben 3, 37073 Göttingen, Germany.
[3]University of Göttingen, Department of Economics, Platz der Göttinger Sieben 3, 37073 Göttingen, Germany; Correspondence to holger.strulik@wiwi.uni-goettingen.de.

Table A1. Health Deficit Items from the HRS Rand Dataset

Dimension	Coding	Wave included
Arthritis	yes= 1, no=0	1
Stroke	yes= 1, no=0, TIA=0.5	1
Diabetes	yes= 1, no=0	1
Lung disease (expect Asthma)	yes= 1, no=0	1
Psychological problem	yes= 1, no=0	1
High Blood Pressure	yes= 1, no=0	1
Heart problem	yes= 1, no=0	1
Cancer	yes=1, no=0	1
Difficulties sitting 2h	no=0, yes=1, can't do=1, don't do=.	1
Difficulties dressing	no=0, yes=1, can't do=1, don't do=.	1
Difficulties bathing/showering w/o help	no=0, yes=1, can't do=1, don't do=.	1
Difficulties walking across room	no=0, yes=1, can't do=1, don't do=.	1
Difficulties lifting 10lbs	no=0, yes=1, can't do=1, don't do=.	1
Difficulties eating	no=0, yes=1, can't do=1, don't do=.	1
Difficulties pushing /pulling large object	no=0, yes=1, can't do=1, don't do=.	1
Difficulties using toilet	no=0, yes=1, can't do=1, don't do=.	2
Difficulties using map	no=0, yes=1, can't do=1, don't do=.	1
Difficulties use a telephone	no=0, yes=1, can't do=1, don't do=.	2
Difficulties kneeing/stoop/crouch	no=0, yes=1, can't do=1, don't do=.	1
Difficulties get in /out of bed	no=0, yes=1, can't do=1, don't do=.	1
Difficulties managing money	no=0, yes=1, can't do=1, don't do=.	2
Difficulties taking medication	no=0, yes=1, can't do=1, don't do=.	2
Difficulties shopping groceries	no=0, yes=1, can't do=1, don't do=.	3
Difficulties preparing hot meals	no=0, yes=1, can't do=1, don't do=.	2
Difficulties walking several blocks	no=0, yes=1, can't do=1, don't do=.	1
Difficulties jogging 1 mile	no=0, yes=1, can't do=1, don't do=.	1
Difficulties walk 1 block	no=0, yes=1, can't do=1, don't do=.	1
Difficulties get up from chair	no=0, yes=1, can't do=1, don't do=.	1
Difficulties climb several flight stair	no=0, yes=1, can't do=1, don't do=.	1
Difficulties climb 1 flight stairs	no=0, yes=1, can't do=1, don't do=.	1
Difficulties picking up a dime	no=0, yes=1, can't do=1, don't do=.	1
Difficulties reach/extend arms up	no=0, yes=1, can't do=1, don't do=.	1
Back problems	yes= 1, no=0	1
Frequency of moderate physical activity	everyday=0, $>$ 1per week=0.25, 1per week=0.5, 1-3 per month=0.75, never=1	7
BMI	BMI$\geq$30 or BMI$\leq$18.5=1, 25$\leq$BMI$<$30=0.5, 18.5$<$BMI$<$25=0	1
Hospital overnight stay	yes= 1, no=0	1
Nursing home stay prev 2 yrs	yes= 1, no=0	1
Living in nursing home at Interview	yes= 1, no=0	3

Table A2. Number of Observations by Year of Birth

Year of Birth	Observations	Year of Birth	Observations	Year of Birth	Observations	Year of Birth	Observations	Year of Birth	Observations	Year of Birth	Observations		
1904	2	1914	886	1924	2,771	1934	6,988	1944	2,559	1954	2,110	1964	13
1905	61	1915	1,076	1925	2,991	1935	7,198	1945	2,439	1955	1,922	1965	1
1906	83	1916	1,145	1926	3,423	1936	7,283	1946	3,452	1956	1,905	1966	4
1907	162	1917	1,479	1927	3,685	1937	7,700	1947	3,419	1957	2,112		
1908	237	1918	1,938	1928	3,935	1938	7,926	1948	2,994	1958	2,230		
1909	346	1919	1,955	1929	3,660	1939	7,911	1949	2,702	1959	2,180		
1910	401	1920	2,398	1930	4,482	1940	8,184	1950	2,920	1960	457		
1911	519	1921	2,608	1931	6,149	1941	8,345	1951	2,828	1961	132		
1912	714	1922	2,880	1932	6,548	1942	4,404	1952	3,287	1962	28		
1913	898	1923	2,771	1933	5,919	1943	2,559	1953	3,181	1963	7		

Table A3. Summary Statistics

Variable	Sample	Obs	Mean	Std. Dev.	Min	Max
		Women				
Health Deficit Index	All	97,321	0.2285	0.1751	0	0.9722
	African American	18,273	0.2726	0.1914	0	0.9722
	Caucasian	76,282	0.2169	0.1685	0	0.9722
log(Health Deficit Index)	All	96,414	-1.8059	0.9249	-7.1389	-0.0282
	African American	18,224	-1.5917	0.8484	-6.0402	-0.0282
	Caucasian	75,442	-1.8608	0.9330	-7.1388	-0.0281
Age	All	97,321	67.8297	10.2679	50	90
	African American	18,273	65.5635	9.7246	50	90
	Caucasian	76,282	68.4958	10.3204	50	90
Year of Birth	All	97,321	1936.194	11.1351	1904	1966
	African American	18,273	1939.495	11.5279	1905	1966
	Caucasian	76,282	1935.205	10.8192	1904	1966
		Men				
Health Deficit Index	All	80,823	0.1866	0.1548	0	0.9697
	African American	12,079	0.2114	0.1754	0	0.9429
	Caucasian	66,341	0.1814	0.1495	0	0.9697
log(Health Deficit Index)	All	80,042	-2.0197	0.9106	-7.1662	-0.0308
	African American	11,970	-1.9091	0.9225	-6.0403	-0.0588
	Caucasian	65,684	-2.0428	0.9058	-7.1663	-0.0308
Age	All	80,823	66.7760	9.6597	50	90
	African American	12,079	65.0423	9.2169	50	90
	Caucasian	66,341	67.2144	9.7112	50	90
Year of Birth	All	80,823	1937.047	10.6306	1904	1964
	African American	12,079	1939.818	11.2095	1905	1964
	Caucasian	66,341	1936.335	10.3627	1904	1964

Figure A.1 Health-Dependent Survival and Survival by Age

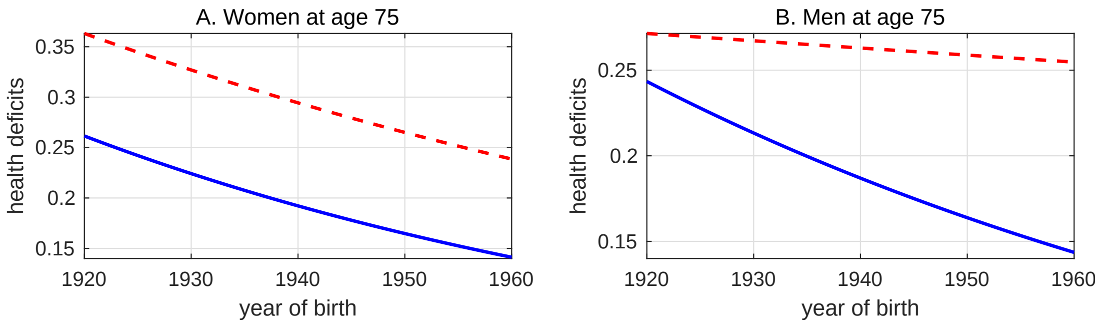

Predicted aging process from estimates columns (5) and (6) of Table 2: Caucasians (blue solid lines) and African Americans (red dashed lines).

Table A4. Panel Results: Women

	(1)	(2)	(3)	(4)	(5)	(6)	(7)	(8)
Age	0.0384***	0.0230**	0.0261***	0.0494***	0.0504***	0.0504***	0.0504***	0.0504***
	(0.00102)	(0.00956)	(0.00531)	(0.00152)	(0.00161)	(0.000484)	(0.00161)	(0.00161)
Midwest	0.0429**	0.0434**	0.00851	0.00484	-0.0408	-0.0409		2.528
	(0.0176)	(0.0176)	(0.0173)	(0.0167)	(0.0430)	(0.0398)		(2.422)
South	0.128***	0.129***	0.0558***	0.0487***	-0.0655**	-0.0655**		0.585
	(0.0196)	(0.0197)	(0.0184)	(0.0180)	(0.0321)	(0.0282)		(2.664)
West	-0.0341	-0.0333	-0.0565***	-0.0626***	-0.0552	-0.0553		8.747***
	(0.0252)	(0.0252)	(0.0200)	(0.0199)	(0.0443)	(0.0427)		(2.582)
1904	-0.0821***	0.500	0.467***	-0.377***	0.887***			
	(0.0281)	(0.302)	(0.159)	(0.0452)	(0.0898)			
1905	-0.445***	0.137	0.0715	-0.773***	0.534***			
	(0.0249)	(0.303)	(0.159)	(0.0424)	(0.0887)			
1906	-0.172***	0.398	0.329**	-0.498***	0.777***			
	(0.0240)	(0.295)	(0.154)	(0.0407)	(0.0862)			
1907	-0.313***	0.236	0.190	-0.612***	0.617***			
	(0.0241)	(0.283)	(0.149)	(0.0408)	(0.0836)			
1908	-0.224***	0.312	0.266*	-0.519***	0.678***			
	(0.0234)	(0.275)	(0.145)	(0.0399)	(0.0813)			
1909	-0.304***	0.210	0.205	-0.553***	0.612***			
	(0.0224)	(0.264)	(0.140)	(0.0382)	(0.0794)			
1910	-0.178***	0.320	0.322**	-0.412***	0.722***			
	(0.0219)	(0.255)	(0.135)	(0.0375)	(0.0770)			
1911	-0.202***	0.276	0.295**	-0.415***	0.682***			
	(0.0211)	(0.245)	(0.130)	(0.0364)	(0.0747)			
1912	-0.118***	0.343	0.320**	-0.364***	0.701***			
	(0.0205)	(0.235)	(0.126)	(0.0355)	(0.0724)			
1913	-0.225***	0.214	0.222*	-0.435***	0.596***			
	(0.0201)	(0.226)	(0.121)	(0.0347)	(0.0698)			
1914	-0.265***	0.152	0.169	-0.457***	0.534***			
	(0.0194)	(0.216)	(0.116)	(0.0334)	(0.0678)			
1915	-0.228***	0.165	0.197*	-0.399***	0.564***			
	(0.0192)	(0.207)	(0.112)	(0.0328)	(0.0655)			
1916	-0.180***	0.195	0.217**	-0.350***	0.591***			
	(0.0181)	(0.198)	(0.107)	(0.0315)	(0.0638)			
1917	-0.240***	0.115	0.147	-0.394***	0.500***			
	(0.0175)	(0.188)	(0.102)	(0.0304)	(0.0609)			
1918	-0.270***	0.0691	0.104	-0.413***	0.447***			
	(0.0168)	(0.179)	(0.0967)	(0.0293)	(0.0585)			
1919	-0.314***	0.00626	0.0186	-0.471***	0.346***			
	(0.0164)	(0.169)	(0.0919)	(0.0285)	(0.0556)			
1920	-0.233***	0.0653	0.0987	-0.362***	0.438***			
	(0.0156)	(0.160)	(0.0872)	(0.0275)	(0.0543)			
1921	-0.186***	0.0986	0.141*	-0.299***	0.440***			
	(0.0147)	(0.150)	(0.0817)	(0.0261)	(0.0503)			
1922	-0.237***	0.0261	0.0590	-0.349***	0.373***			
	(0.0145)	(0.141)	(0.0773)	(0.0258)	(0.0488)			
1923	-0.253***	-0.00786	0.0166	-0.365***	0.336***			
	(0.0140)	(0.132)	(0.0727)	(0.0250)	(0.0474)			
1924	-0.232***	-0.00765	0.0208	-0.335***	0.362***			
	(0.0141)	(0.123)	(0.0689)	(0.0252)	(0.0472)			
1925	-0.184***	0.0253	0.0599	-0.271***	0.381***			
	(0.0132)	(0.114)	(0.0640)	(0.0239)	(0.0441)			
1926	-0.181***	0.0106	0.00912	-0.295***	0.340***			
	(0.0131)	(0.104)	(0.0594)	(0.0235)	(0.0428)			
1927	-0.192***	-0.0186	-0.00906	-0.288***	0.306***			
	(0.0119)	(0.0945)	(0.0542)	(0.0221)	(0.0402)			
1928	-0.208***	-0.0493	-0.0373	-0.293***	0.261***			
	(0.0112)	(0.0855)	(0.0492)	(0.0210)	(0.0376)			
1929	-0.0915***	0.0485	0.0320	-0.197***	0.311***			
	(0.0102)	(0.0756)	(0.0441)	(0.0199)	(0.0342)			
1930	-0.158***	-0.0343	-0.0509	-0.254***	0.197***			
	(0.00907)	(0.0658)	(0.0386)	(0.0183)	(0.0305)			
1931	-0.0645***	0.0301	0.0584*	-0.0890***	0.157***			
	(0.00588)	(0.0576)	(0.0320)	(0.0136)	(0.0170)			
1932	-0.0675***	0.0101	0.0621**	-0.0585***	0.155***			
	(0.00491)	(0.0477)	(0.0269)	(0.0128)	(0.0147)			
1933	-0.0444***	0.0183	0.0504**	-0.0489***	0.127***			
	(0.00424)	(0.0383)	(0.0220)	(0.0121)	(0.0124)			
1934	-0.0191***	0.0282	0.0750***	-0.000615	0.117***			
	(0.00326)	(0.0286)	(0.0167)	(0.0112)	(0.00859)			
1935	0.00426	0.0360*	0.0557***	0.000447	0.0899***			
	(0.00271)	(0.0192)	(0.0121)	(0.0109)	(0.00717)			
1936	-0.0110***	0.00489	0.0117	-0.0172*	0.0222***			
	(0.00246)	(0.0101)	(0.00761)	(0.0103)	(0.00445)			
1938	0.118***	0.102***	0.0743***	0.0944***	0.0476***			
	(0.00174)	(0.00958)	(0.00617)	(0.00955)	(0.00493)			
1939	0.0597***	0.0286	0.0193*	0.0609***	-0.0415***			
	(0.00239)	(0.0191)	(0.0107)	(0.00937)	(0.00798)			
1940	0.0842***	0.0390	0.0393**	0.102***	-0.0460***			
	(0.00308)	(0.0287)	(0.0157)	(0.00948)	(0.0108)			
1941	0.129***	0.0677*	0.0390*	0.127***	-0.0691***			
	(0.00398)	(0.0381)	(0.0207)	(0.00988)	(0.0139)			
1942	0.128***	0.0578	0.00971	0.108***	-0.0637***			
	(0.00391)	(0.0470)	(0.0249)	(0.00962)	(0.0125)			
1943	0.185***	0.108*	0.100***	0.201***	0.0531***			
	(0.00342)	(0.0575)	(0.0300)	(0.00949)	(0.0112)			
1944	0.137***	0.0436	-0.0138	0.112***	-0.0550***			
	(0.00448)	(0.0677)	(0.0348)	(0.00999)	(0.0117)			
1945	0.0647***	-0.0428	-0.106***	0.0436***	-0.164***			
	(0.00466)	(0.0766)	(0.0397)	(0.00998)	(0.0149)			
1946	0.105***	-0.0211	-0.0641	0.115***	-0.120***			
	(0.00550)	(0.0874)	(0.0451)	(0.0104)	(0.0164)			
1947	0.151***	0.0109	-0.0518	0.147***	-0.134***			
	(0.00631)	(0.0956)	(0.0499)	(0.0108)	(0.0197)			
1948	0.112***	-0.0576	-0.102*	0.144***	-0.0279**			
	(0.00495)	(0.105)	(0.0529)	(0.00989)	(0.0124)			
1949	0.123***	-0.0697	-0.151***	0.126***	-0.0558***			
	(0.00531)	(0.114)	(0.0577)	(0.0100)	(0.0136)			
1950	0.238***	0.0306	-0.0383	0.263***	0.0286*			
	(0.00575)	(0.123)	(0.0627)	(0.0103)	(0.0164)			
1951	0.0983***	-0.124	-0.186***	0.137***	-0.143***			
	(0.00674)	(0.132)	(0.0677)	(0.0111)	(0.0197)			
1952	0.135***	-0.104	-0.163**	0.185***	-0.153***			
	(0.00784)	(0.142)	(0.0732)	(0.0121)	(0.0235)			
1953	0.264***	0.00930	-0.0660	0.305***	-0.0788***			
	(0.00888)	(0.152)	(0.0786)	(0.0130)	(0.0265)			
1954	0.158***	-0.105	-0.181**	0.202***	-0.132***			
	(0.00814)	(0.160)	(0.0829)	(0.0122)	(0.0235)			
1955	0.286***	0.00939	-0.0656	0.337***	-0.0181			
	(0.00815)	(0.170)	(0.0882)	(0.0124)	(0.0243)			
1956	0.281***	-0.00984	-0.0944	0.330***	-0.0722***			
	(0.00940)	(0.179)	(0.0932)	(0.0136)	(0.0273)			
1957	0.298***	-0.00884	-0.109	0.339***	-0.111***			
	(0.0103)	(0.188)	(0.0983)	(0.0147)	(0.0311)			
1958	0.263***	-0.0592	-0.160	0.312***	-0.193***			
	(0.0111)	(0.198)	(0.104)	(0.0158)	(0.0348)			
1959	0.360***	0.0230	-0.0703	0.424***	-0.132***			
	(0.0120)	(0.208)	(0.109)	(0.0168)	(0.0380)			
1960	0.0371***	-0.313	-0.386***	0.127***	-0.470***			
	(0.0132)	(0.216)	(0.113)	(0.0182)	(0.0408)			
1961	0.596***	0.242	0.178	0.696***	0.0936**			
	(0.0132)	(0.225)	(0.118)	(0.0178)	(0.0410)			
1962	0.649***	0.294	-0.0876	0.431***	-0.141***			
	(0.0145)	(0.233)	(0.123)	(0.0179)	(0.0398)			
1963	0.472***	0.119	-0.173	0.352***	-0.214***			
	(0.0148)	(0.247)	(0.130)	(0.0187)	(0.0384)			
1964	1.116***	0.744***	0.594***	1.149***	0.501***			
	(0.0156)	(0.250)	(0.132)	(0.0201)	(0.0459)			
Wave 2		-0.0989***	-0.169***					
		(0.0191)	(0.0155)					
Wave 3		-0.222***	-0.257***					
		(0.0374)	(0.0321)					
Wave 4		-0.180***	-0.218***					
		(0.0617)	(0.0363)					
Wave 5		-0.120	-0.145***					
		(0.0798)	(0.0444)					
Wave 6		-0.0854	-0.0893*					
		(0.101)	(0.0505)					
Wave 7		0.0231	0.0729					
		(0.117)	(0.0597)					
Wave 8		0.0559	0.117*					
		(0.132)	(0.0664)					
Wave 9		0.0751	0.154**					
		(0.153)	(0.0765)					
Wave 10		0.140	0.227**					
		(0.172)	(0.0882)					
Wave 11		0.135	0.225**					
		(0.189)	(0.0965)					
Wave 12		0.137	0.240**					
		(0.208)	(0.106)					
Wave 13		0.138	0.254**					
		(0.231)	(0.120)					
Mean Age					-0.0520***	-0.0419***	-0.0419***	-0.0419***
					(0.00459)	(0.00192)	(0.00417)	(0.00416)
Year of birth						-0.00989***		
						(0.00139)		
Northeast × Year of birth							-0.00986***	-0.00866***
							(0.00231)	(0.00236)
Midwest × Year of birth							-0.00988***	-0.00999***
							(0.00231)	(0.00242)
South × Year of birth							-0.00990***	-0.00900***
							(0.00231)	(0.00250)
West × Year of birth							-0.00989***	-0.0132***
							(0.00232)	(0.00248)
Constant	-4.476***	-3.451***	-3.588***	-5.088***	-1.868***	16.72***	16.67***	14.35***
	(0.0699)	(0.523)	(0.295)	(0.0995)	(0.219)	(2.812)	(4.667)	(4.740)
Method	OLS	OLS	RE	RE	Mundlak	Mundlak	Mundlak	Mundlak + Region Dummy
N	96414	96414	96414	96414	96414	96414	96414	96414

Standard errors clustered at the year of birth level in parentheses.

Dependent variable is the natural logarithm of the health deficit index. The baseline for wave and region dummy variables are Wave 1 and Northeast respectively. The (time) means of the time changing variables are included in columns (5) to (8).

* $p < 0.10$, ** $p < 0.05$, *** $p < 0.01$

Table A5. Panel Results: Men

	(1)	(2)	(3)	(4)	(5)	(6)	(7)	(8)
Age	0.0424***	0.0181	0.0248***	0.0550***	0.0565***	0.0565***	0.0565***	0.0565***
	(0.000678)	(0.0111)	(0.00604)	(0.00114)	(0.00123)	(0.00123)	(0.00123)	(0.00122)
Midwest	0.0164	0.0155	-0.00455	-0.00416	-0.0331	-0.0331		1.285
	(0.0223)	(0.0224)	(0.0193)	(0.0193)	(0.0406)	(0.0406)		(2.952)
South	0.0946***	0.0952***	0.0150	0.0138	-0.112***	-0.112***		-3.379
	(0.0174)	(0.0174)	(0.0192)	(0.0193)	(0.0362)	(0.0362)		(2.720)
West	-0.0557**	-0.0560**	-0.0976***	-0.0955***	-0.115**	-0.115**		-1.764
	(0.0218)	(0.0218)	(0.0208)	(0.0207)	(0.0511)	(0.0511)		(2.978)
1904	-0.541***	0.292	0.143	-0.892***	0.464***			
	(0.0172)	(0.347)	(0.189)	(0.0309)	(0.0598)			
1905	-0.661***	0.171	-0.0138	-1.047***	0.345***			
	(0.0169)	(0.348)	(0.187)	(0.0302)	(0.0573)			
1906	-0.477***	0.334	0.150	-0.860***	0.498***			
	(0.0166)	(0.339)	(0.183)	(0.0287)	(0.0558)			
1907	-0.527***	0.257	0.127	-0.849***	0.468***			
	(0.0163)	(0.328)	(0.178)	(0.0280)	(0.0544)			
1908	-0.249***	0.519	0.360**	-0.594***	0.698***			
	(0.0158)	(0.318)	(0.173)	(0.0275)	(0.0538)			
1909	-0.515***	0.221	0.0872	-0.829***	0.422***			
	(0.0151)	(0.306)	(0.166)	(0.0266)	(0.0521)			
1910	-0.252***	0.463	0.363**	-0.528***	0.684***			
	(0.0147)	(0.297)	(0.161)	(0.0258)	(0.0501)			
1911	-0.223***	0.467	0.336**	-0.523***	0.648***			
	(0.0142)	(0.286)	(0.154)	(0.0251)	(0.0486)			
1912	-0.352***	0.312	0.233	-0.595***	0.547***			
	(0.0140)	(0.275)	(0.149)	(0.0243)	(0.0471)			
1913	-0.356***	0.278	0.223	-0.571***	0.524***			
	(0.0134)	(0.264)	(0.143)	(0.0234)	(0.0456)			
1914	-0.278***	0.327	0.274**	-0.483***	0.571***			
	(0.0130)	(0.253)	(0.137)	(0.0224)	(0.0435)			
1915	-0.383***	0.195	0.202	-0.521***	0.490***			
	(0.0125)	(0.242)	(0.131)	(0.0214)	(0.0417)			
1916	-0.212***	0.340	0.287**	-0.406***	0.569***			
	(0.0127)	(0.231)	(0.125)	(0.0205)	(0.0395)			
1917	-0.304***	0.219	0.186	-0.470***	0.472***			
	(0.0121)	(0.220)	(0.119)	(0.0198)	(0.0388)			
1918	-0.283***	0.210	0.0882	-0.532***	0.365***			
	(0.0113)	(0.209)	(0.113)	(0.0190)	(0.0367)			
1919	-0.290***	0.178	0.158	-0.430***	0.421***			
	(0.0108)	(0.198)	(0.107)	(0.0180)	(0.0351)			
1920	-0.189***	0.253	0.198*	-0.358***	0.457***			
	(0.0102)	(0.187)	(0.101)	(0.0173)	(0.0336)			
1921	-0.263***	0.152	0.145	-0.378***	0.386***			
	(0.00976)	(0.176)	(0.0952)	(0.0164)	(0.0315)			
1922	-0.177***	0.209	0.188**	-0.301***	0.449***			
	(0.00951)	(0.165)	(0.0896)	(0.0158)	(0.0309)			
1923	-0.154***	0.206	0.179**	-0.278***	0.384***			
	(0.00857)	(0.154)	(0.0830)	(0.0140)	(0.0272)			
1924	-0.186***	0.143	0.120	-0.301***	0.422***			
	(0.00885)	(0.144)	(0.0789)	(0.0154)	(0.0302)			
1925	-0.183***	0.121	0.106	-0.282***	0.394***			
	(0.00857)	(0.133)	(0.0727)	(0.0143)	(0.0276)			
1926	-0.148***	0.129	0.0553	-0.298***	0.327***			
	(0.00787)	(0.122)	(0.0664)	(0.0132)	(0.0257)			
1927	-0.0559***	0.196*	0.143**	-0.182***	0.382***			
	(0.00708)	(0.111)	(0.0606)	(0.0120)	(0.0231)			
1928	0.0152**	0.240**	0.190***	-0.0972***	0.401***			
	(0.00643)	(0.100)	(0.0546)	(0.0105)	(0.0202)			
1929	-0.0569***	0.141	0.104**	-0.147***	0.279***			
	(0.00563)	(0.0883)	(0.0480)	(0.00896)	(0.0171)			
1930	-0.121***	0.0527	0.0261	-0.195***	0.174***			
	(0.00502)	(0.0773)	(0.0424)	(0.00776)	(0.0151)			
1931	-0.0302***	0.116*	0.113***	-0.0675***	0.202***			
	(0.00378)	(0.0661)	(0.0362)	(0.00566)	(0.0112)			
1932	-0.108***	0.0149	0.0519*	-0.100***	0.117***			
	(0.00290)	(0.0555)	(0.0301)	(0.00463)	(0.00878)			
1933	0.0639***	0.162***	0.185***	0.0624***	0.222***			
	(0.00233)	(0.0444)	(0.0243)	(0.00335)	(0.00641)			
1934	0.0913***	0.165***	0.179***	0.0864***	0.209***			
	(0.00171)	(0.0331)	(0.0180)	(0.00261)	(0.00505)			
1935	0.0150***	0.0648***	0.0504***	-0.0140***	0.0869***			
	(0.00138)	(0.0223)	(0.0121)	(0.00248)	(0.00434)			
1936	0.0430***	0.0691***	0.0916***	0.0583***	0.111***			
	(0.000604)	(0.0117)	(0.00629)	(0.00116)	(0.00211)			
1938	0.107***	0.0828***	0.104***	0.134***	0.0932***			
	(0.000698)	(0.0113)	(0.00617)	(0.00100)	(0.00199)			
1939	0.118***	0.0691***	0.0668***	0.126***	0.0211***			
	(0.00159)	(0.0222)	(0.0120)	(0.00225)	(0.00433)			
1940	0.0799***	0.00655	0.00866	0.0989***	-0.0387***			
	(0.00208)	(0.0335)	(0.0183)	(0.00284)	(0.00576)			
1941	0.123***	0.0272	0.0510**	0.169***	-0.0141*			
	(0.00260)	(0.0441)	(0.0239)	(0.00405)	(0.00784)			
1942	0.0529***	-0.0604	-0.0758**	0.0578***	-0.0164***			
	(0.00174)	(0.0549)	(0.0298)	(0.00172)	(0.00320)			
1943	0.147***	0.0105	-0.0199	0.142***	0.0703***			
	(0.00191)	(0.0666)	(0.0360)	(0.00156)	(0.00286)			
1944	0.134***	-0.0269	-0.00135	0.191***	0.0567***			
	(0.00247)	(0.0777)	(0.0423)	(0.00286)	(0.00569)			
1945	0.152***	-0.0311	-0.0401	0.181***	0.0282***			
	(0.00318)	(0.0880)	(0.0479)	(0.00326)	(0.00613)			
1946	0.127***	-0.0857	-0.124**	0.134***	-0.0649***			
	(0.00347)	(0.101)	(0.0542)	(0.00444)	(0.00822)			
1947	0.120***	-0.115	-0.129**	0.155***	-0.0853***			
	(0.00402)	(0.111)	(0.0603)	(0.00513)	(0.00993)			
1948	0.220***	-0.0588	-0.0888	0.255***	0.128***			
	(0.00271)	(0.122)	(0.0664)	(0.00289)	(0.00540)			
1949	0.141***	-0.164	-0.233***	0.142***	-0.0281***			
	(0.00342)	(0.133)	(0.0726)	(0.00396)	(0.00744)			
1950	0.286***	-0.0437	-0.0946	0.312***	0.0922***			
	(0.00385)	(0.144)	(0.0786)	(0.00485)	(0.00920)			
1951	0.242***	-0.111	-0.151*	0.284***	0.0119			
	(0.00461)	(0.155)	(0.0845)	(0.00629)	(0.0117)			
1952	0.116***	-0.261	-0.303***	0.162***	-0.161***			
	(0.00519)	(0.166)	(0.0903)	(0.00712)	(0.0134)			
1953	0.239***	-0.161	-0.170*	0.322***	-0.0348**			
	(0.00584)	(0.177)	(0.0966)	(0.00810)	(0.0152)			
1954	0.306***	-0.115	-0.174*	0.338***	0.0411***			
	(0.00500)	(0.188)	(0.103)	(0.00652)	(0.0122)			
1955	0.363***	-0.0809	-0.122	0.418***	0.0845***			
	(0.00529)	(0.200)	(0.110)	(0.00748)	(0.0141)			
1956	0.332***	-0.137	-0.183	0.388***	-0.000378			
	(0.00602)	(0.211)	(0.115)	(0.00862)	(0.0162)			
1957	0.316***	-0.176	-0.232*	0.368***	-0.0687***			
	(0.00648)	(0.222)	(0.121)	(0.00968)	(0.0184)			
1958	0.318***	-0.198	-0.247*	0.382***	-0.111***			
	(0.00734)	(0.232)	(0.127)	(0.0107)	(0.0204)			
1959	0.358***	-0.183	-0.220*	0.440***	-0.104***			
	(0.00800)	(0.244)	(0.133)	(0.0120)	(0.0226)			
1960	0.481***	-0.0758	-0.0648	0.609***	0.0356			
	(0.00930)	(0.253)	(0.138)	(0.0125)	(0.0229)			
1961	-0.119***	-0.692**	-0.701***	-0.00459	-0.603***			
	(0.00893)	(0.263)	(0.144)	(0.0133)	(0.0248)			
1962	0.648***	0.0631	-0.0355	0.675***	0.0464*			
	(0.0121)	(0.278)	(0.151)	(0.0139)	(0.0251)			
1963	0.628***	0.0234	0.0152	0.755***	0.0914***			
	(0.0130)	(0.300)	(0.164)	(0.0147)	(0.0260)			
1964	0.443***	-0.159	-0.294*	0.439***	-0.180***			
	(0.00915)	(0.296)	(0.163)	(0.0137)	(0.0255)			
Wave 2		-0.0722**	-0.125***					
		(0.0294)	(0.0157)					
Wave 3		-0.106**	-0.131***					
		(0.0502)	(0.0304)					
Wave 4		-0.0552	-0.0701*					
		(0.0705)	(0.0386)					
Wave 5		0.00161	-0.00102					
		(0.0890)	(0.0462)					
Wave 6		0.0613	0.0793					
		(0.112)	(0.0593)					
Wave 7		0.194	0.256***					
		(0.134)	(0.0695)					
Wave 8		0.215	0.282***					
		(0.157)	(0.0840)					
Wave 9		0.271	0.347***					
		(0.177)	(0.0947)					
Wave 10		0.368*	0.446***					
		(0.206)	(0.109)					
Wave 11		0.376*	0.450***					
		(0.223)	(0.121)					
Wave 12		0.388	0.470***					
		(0.245)	(0.135)					
Wave 13		0.408	0.497***					
		(0.272)	(0.149)					
Mean Age					-0.0546***	-0.0461***	-0.0461***	-0.0462***
					(0.00290)	(0.00273)	(0.00273)	(0.00272)
Year of birth						-0.00835***		
						(0.00164)		
Northeast × Year of birth							-0.00831***	-0.00910***
							(0.00164)	(0.00183)
Midwest × Year of birth							-0.00833***	-0.00978***
							(0.00164)	(0.00178)
South × Year of birth							-0.00837***	-0.00742***
							(0.00164)	(0.00180)
West × Year of birth							-0.00837***	-0.00825***
							(0.00164)	(0.00195)
Constant	-4.929***	-3.469***	-3.818***	-5.628***	-2.303***	13.46***	13.38***	14.93***
	(0.0430)	(0.607)	(0.330)	(0.0762)	(0.143)	(3.299)	(3.298)	(3.640)
Method	OLS	OLS	RE	RE	Mundlak	Mundlak	Mundlak	Mundlak + Region Dummy
N	80042	80042	80042	80042	80042	80042	80042	80042

Standard errors clustered at the year of birth level in parentheses.
Dependent variable is the natural logarithm of the health deficit index. The baseline for wave and region dummy variables are Wave 1 and Northeast respectively. The (time) means of the time changing variables are included in columns (5) to (8).
* $p < 0.10$, ** $p < 0.05$, *** $p < 0.01$

Table A6. Robustness FE & Mundlak Present next wave, Women

	(1)	(2)	(3)	(4)	(5)	(6)
Age	0.0494***	0.0455***	0.0505***	0.0492***	0.0454***	0.0502***
	(0.00148)	(0.00127)	(0.00164)	(0.00147)	(0.00127)	(0.00163)
Midwest	-0.0403	0.150*	-0.0762	-0.0405	0.150*	-0.0766
	(0.0429)	(0.0804)	(0.0498)	(0.0428)	(0.0804)	(0.0497)
South	-0.0640*	-0.0232	-0.0680*	-0.0642**	-0.0229	-0.0684*
	(0.0321)	(0.0554)	(0.0363)	(0.0320)	(0.0555)	(0.0362)
West	-0.0540	0.0180	-0.0745	-0.0544	0.0181	-0.0751
	(0.0441)	(0.125)	(0.0474)	(0.0439)	(0.125)	(0.0472)
Year of birth				-0.00924***	-0.0103***	-0.0143***
				(0.00213)	(0.00264)	(0.00210)
Mean Age				-0.0398***	-0.0429***	-0.0428***
				(0.00375)	(0.00430)	(0.00382)
Responded next wave	-0.0389***	-0.00834	-0.0493***	-0.0461***	-0.00940	-0.0568***
	(0.0105)	(0.0118)	(0.0111)	(0.0105)	(0.0115)	(0.0111)
Constant	-5.085***	-4.585***	-5.224***	15.44***	18.31***	25.24***
	(0.0980)	(0.0980)	(0.110)	(4.304)	(5.342)	(4.230)
Sample	All	African American	Caucasian	All	African American	Caucasian
N	96414	18224	75442	96414	18224	75442

Standard errors clustered at the year of birth level in parentheses. The (time) means of the time changing variables are included in columns (4) to (6).
* $p < 0.10$, ** $p < 0.05$, *** $p < 0.01$

Table A7. Robustness FE & Mundlak Present next wave, Men

	(1)	(2)	(3)	(4)	(5)	(6)
Age	0.0549***	0.0540***	0.0550***	0.0546***	0.0536***	0.0547***
	(0.00107)	(0.00135)	(0.00112)	(0.00106)	(0.00133)	(0.00110)
Midwest	-0.0328	-0.173	-0.00694	-0.0327	-0.167	-0.00744
	(0.0401)	(0.117)	(0.0444)	(0.0400)	(0.118)	(0.0445)
South	-0.109***	-0.173**	-0.0963**	-0.109***	-0.174**	-0.0963**
	(0.0359)	(0.0801)	(0.0417)	(0.0359)	(0.0785)	(0.0417)
West	-0.115**	-0.221**	-0.104*	-0.113**	-0.203*	-0.105*
	(0.0502)	(0.107)	(0.0545)	(0.0505)	(0.105)	(0.0547)
Year of birth				-0.00745***	-0.00144	-0.0118***
				(0.00148)	(0.00223)	(0.00160)
Mean Age				-0.0429***	-0.0378***	-0.0461***
				(0.00230)	(0.00301)	(0.00251)
Responded next wave	-0.0604***	-0.0212	-0.0700***	-0.0702***	-0.0303*	-0.0787***
	(0.0130)	(0.0154)	(0.0132)	(0.0128)	(0.0158)	(0.0129)
Constant	-5.568***	-5.252***	-5.628***	11.69***	-0.146	20.34***
	(0.0842)	(0.0908)	(0.0900)	(2.975)	(4.473)	(3.226)
Sample	All	African American	Caucasian	All	African American	Caucasian
N	80042	11970	65684	80042	11970	65684

Standard errors clustered at the year of birth level in parentheses. The (time) means of the time changing variables are included in columns (4) to (6).
* $p < 0.10$, ** $p < 0.05$, *** $p < 0.01$

Table A8. Linear Results Women, Age Restriction 50-85

	(1)	(2)	(3)	(4)	(5)	(6)
Age	0.0485***	0.0448***	0.0495***	0.0485***	0.0449***	0.0496***
	(0.00125)	(0.00119)	(0.00139)	(0.00127)	(0.00119)	(0.00141)
Midwest	-0.0245	0.145*	-0.0596	-0.0225	0.149*	-0.0577
	(0.0433)	(0.0816)	(0.0505)	(0.0431)	(0.0816)	(0.0501)
South	-0.0571*	-0.0233	-0.0617*	-0.0561*	-0.0231	-0.0603*
	(0.0316)	(0.0510)	(0.0364)	(0.0315)	(0.0514)	(0.0363)
West	-0.0417	0.0793	-0.0673	-0.0399	0.0782	-0.0649
	(0.0458)	(0.114)	(0.0494)	(0.0455)	(0.114)	(0.0491)
Year of birth				-0.0112***	-0.0112***	-0.0169***
				(0.00245)	(0.00268)	(0.00253)
Mean Age				-0.0433***	-0.0435***	-0.0470***
				(0.00400)	(0.00427)	(0.00411)
Constant	-5.040***	-4.538***	-5.189***	19.58***	20.09***	30.55***
	(0.0832)	(0.0920)	(0.0940)	(4.939)	(5.414)	(5.086)
Sample	All	African American	Caucasian	All	African American	Caucasian
N	91386	17609	71102	91386	17609	71102

Standard errors clustered at the year of birth level in parentheses. The (time) means of the time changing variables are included in columns (4) to (6).
* $p < 0.10$, ** $p < 0.05$, *** $p < 0.01$

Table A9. Linear Results Men, Age Restriction 50-85

	(1)	(2)	(3)	(4)	(5)	(6)
Age	0.0554***	0.0537***	0.0557***	0.0554***	0.0536***	0.0557***
	(0.00107)	(0.00140)	(0.00112)	(0.00108)	(0.00140)	(0.00113)
Midwest	-0.0361	-0.159	-0.0116	-0.0365	-0.155	-0.0126
	(0.0397)	(0.118)	(0.0441)	(0.0397)	(0.119)	(0.0442)
South	-0.103***	-0.150*	-0.0921**	-0.105***	-0.153**	-0.0933**
	(0.0351)	(0.0794)	(0.0414)	(0.0351)	(0.0777)	(0.0414)
West	-0.117**	-0.181*	-0.110**	-0.117**	-0.167*	-0.111**
	(0.0504)	(0.102)	(0.0546)	(0.0508)	(0.0996)	(0.0548)
Year of birth				-0.00939***	-0.00234	-0.0142***
				(0.00178)	(0.00235)	(0.00192)
Mean Age				-0.0471***	-0.0401***	-0.0508***
				(0.00276)	(0.00327)	(0.00299)
Constant	-5.631***	-5.254***	-5.710***	15.60***	1.716	25.21***
	(0.0846)	(0.0957)	(0.0909)	(3.583)	(4.700)	(3.876)
Sample	All	African American	Caucasian	All	African American	Caucasian
N	77308	11681	63265	77308	11681	63265

Standard errors clustered at the year of birth level in parentheses.The (time) means of the time changing variables are included in columns (4) to (6).
* $p < 0.10$, ** $p < 0.05$, *** $p < 0.01$

Table A10. Linear Results Women, without upper Age Restriction

	(1)	(2)	(3)	(4)	(5)	(6)
Age	0.0518***	0.0465***	0.0532***	0.0518***	0.0465***	0.0531***
	(0.00183)	(0.00136)	(0.00200)	(0.00183)	(0.00136)	(0.00201)
Midwest	-0.0371	0.154*	-0.0719	-0.0374	0.154*	-0.0724
	(0.0418)	(0.0827)	(0.0484)	(0.0417)	(0.0827)	(0.0483)
South	-0.0628*	-0.0217	-0.0649*	-0.0632**	-0.0215	-0.0655*
	(0.0317)	(0.0560)	(0.0359)	(0.0316)	(0.0560)	(0.0357)
West	-0.0501	0.0224	-0.0692	-0.0506	0.0225	-0.0699
	(0.0442)	(0.126)	(0.0482)	(0.0441)	(0.126)	(0.0481)
Year of birth				-0.0105***	-0.0105***	-0.0162***
				(0.00235)	(0.00261)	(0.00240)
Mean Age				-0.0414***	-0.0421***	-0.0454***
				(0.00418)	(0.00414)	(0.00434)
Constant	-5.294***	-4.675***	-5.467***	17.73***	18.47***	28.87***
	(0.126)	(0.103)	(0.141)	(4.736)	(5.267)	(4.811)
Sample	All	African American	Caucasian	All	African American	Caucasian
N	98914	18591	77545	98914	18591	77545

Standard errors clustered at the year of birth level in parentheses. The (time) means of the time changing variables are included in columns (4) to (6).
* $p < 0.10$, ** $p < 0.05$, *** $p < 0.01$

Table A11. Linear Results Men, without upper Age restriction

	(1)	(2)	(3)	(4)	(5)	(6)
Age	0.0573***	0.0552***	0.0576***	0.0572***	0.0551***	0.0575***
	(0.00133)	(0.00154)	(0.00138)	(0.00133)	(0.00155)	(0.00138)
Midwest	-0.0315	-0.176	-0.00483	-0.0314	-0.171	-0.00532
	(0.0414)	(0.119)	(0.0460)	(0.0414)	(0.119)	(0.0461)
South	-0.108***	-0.182**	-0.0944**	-0.109***	-0.183**	-0.0946**
	(0.0360)	(0.0813)	(0.0414)	(0.0361)	(0.0798)	(0.0415)
West	-0.120**	-0.270**	-0.102*	-0.119**	-0.253**	-0.103*
	(0.0504)	(0.114)	(0.0552)	(0.0508)	(0.114)	(0.0554)
Year of birth				-0.00884***	-0.00189	-0.0136***
				(0.00169)	(0.00228)	(0.00182)
Mean Age				-0.0460***	-0.0388***	-0.0498***
				(0.00282)	(0.00326)	(0.00302)
Constant	-5.782***	-5.346***	-5.872***	14.37***	0.669	23.80***
	(0.101)	(0.101)	(0.108)	(3.395)	(4.571)	(3.653)
Sample	All	African American	Caucasian	All	African American	Caucasian
N	81034	12083	66552	81034	12083	66552

Standard errors clustered at the year of birth level in parentheses. The (time) means of the time changing variables are included in columns (4) to (6).
* $p < 0.10$, ** $p < 0.05$, *** $p < 0.01$

Table A12. Nonlinear least square

	Men	Women
A	0.0572***	0.116***
	(0.0111)	(0.00775)
R	0.0118***	0.00601***
	(0.00353)	(0.00174)
alpha	0.0350***	0.0417***
	(0.00307)	(0.00309)
Observations	78088	92292
R^2	0.073	0.061
Adjusted R^2	0.073	0.061

Robust Standard errors in parentheses, age restriction 50-85, with initial values (M:(A 0.02 R 0.0031198 alpha 0.043), W:(A 0.02 R 0.0097548 alpha 0.031)). * $p < 0.10$, ** $p < 0.05$, *** $p < 0.01$

Table A13. Nonlinear least square

	Men	Women
A	0.0506***	0.136***
	(0.00884)	(0.00295)
R	0.0140***	0.00221***
	(0.00289)	(0.000299)
alpha	0.0333***	0.0528***
	(0.00204)	(0.00142)
Observations	81816	99821
R^2	0.101	0.123
Adjusted R^2	0.101	0.123

Robust Standard errors in parentheses, at least 50 years old without upper age restriction, with initial values (M:(A 0.02 R 0.0031198 alpha 0.043), W:(A 0.02 R 0.0097548 alpha 0.031)). * $p < 0.10$, ** $p < 0.05$, *** $p < 0.01$

Table A14. Nonlinear least square

	Men	Women
A	0.0520***	0.135***
	(0.00987)	(0.00552)
R	0.0136***	0.00214***
	(0.00308)	(0.000562)
alpha	0.0335***	0.0533***
	(0.00218)	(0.00278)
Observations	41	41
R^2	0.995	0.993
Adjusted R^2	0.995	0.993

Standard errors in parentheses. One-year binning, 50 - 90 age cutoff, with initial values (M:(A 0.02 R 0.0031198 alpha 0.043), W:(A 0.02 R 0.0097548 alpha 0.031)). * $p < 0.10$, ** $p < 0.05$, *** $p < 0.01$
